# Resistance to immune checkpoint inhibitors in KRAS-mutant non-small cell lung cancer

**DOI:** 10.20517/cdr.2021.102

**Published:** 2022-02-08

**Authors:** Yunchang Li, Lanlin Hu, Xinhao Peng, Huasheng Xu, Bo Tang, Chuan Xu

**Affiliations:** ^1^Integrative Cancer Center and Cancer Clinical Research Center, Sichuan Cancer Hospital and Institute, Sichuan Cancer Center, School of Medicine, University of Electronic Science and Technology of China, Chengdu 610042, Sichuan, China.; ^2^Collaborative Innovation Centre of Regenerative Medicine and Medical BioResource Development and Application, Guangxi Medical University, Nanning 530021, Guangxi, China.; ^#^Authors contributed equally.

**Keywords:** Non-small cell lung cancer, KRAS mutation, immune checkpoints inhibitors, tumor microenvironments, cancer metabolism.

## Abstract

Non-small cell lung cancer (NSCLC) patients with Kirsten rat sarcoma viral oncogene homolog (KRAS) mutation are associated with significant clinical heterogeneity and a poor prognosis to standard NSCLC therapies such as surgical resection, radiotherapy, chemotherapies, and targeted medicines. However, the application of immune checkpoints inhibitors (ICIs) has dramatically altered the therapeutic pattern of NSCLC management. Clinical studies have indicated that some KRAS-mutant NSCLC patients could benefit from ICIs; however, the responses in some patients are still poor. This review intends to elucidate the mechanisms of resistance to immunotherapy in KRAS-driven NSCLC and highlight the TME functions altered by immunoinhibitors, immunostimulators, and cancer metabolism. These metabolic pathways could potentially be promising approaches to overcome immunotherapy resistance.

## INTRODUCTION

Lung cancer is the most commonly diagnosed cancer and the leading cause of cancer-related deaths worldwide^[1]^. According to Global Cancer Statistics 2020, lung cancer is estimated to represent 11.4% of new cancer cases and 18.0% of cancer-related deaths^[1]^. There are two main histological types of lung cancer: non-small cell lung cancer (NSCLC) and small cell lung cancer (SCLC)^[2]^. About 85% of lung cancer is NSCLC, which comprises lung adenocarcinoma (LUAD), lung squamous cell carcinoma, and large cell neuroendocrine carcinoma. SCLC accounts for the remaining 15%^[3]^. Despite inducements such as smoking, genetic mutations are the leading causes of lung cancer. The most common driver mutations in NSCLC involve Kirsten rat sarcoma viral oncogene homolog (KRAS), epidermal growth factor receptor (EGFR), and anaplastic lymphoma kinase^[4]^.

KRAS, a member of the RAS family, was one of the first oncogenes identified in NSCLC^[5]^. Since KRAS mutation is reported to be associated with resistance to multiple therapies and poor prognosis in NSCLC, several preclinical and clinical studies have investigated effective therapies, including immunotherapy and targeted therapy^[6,7]^. In 2021, AMG510, specifically targeting KRAS^G12C^, was approved by the FDA as the orphan drug to treat the NSCLC with KRAS^G12C^^[8]^. Other inhibitors, peptides, and tumor vaccines are under preclinical and clinical studies, including MRTX849 targeting KRAS^G12C^, MRTX1133 targeting KRAS^G12D^, 12VC1 targeting KRAS^G12C/V^, mRNA-5671 targeting KRAS^G12C/D/V^, *etc.*^[9]^. In addition to targeted therapy, immunotherapies have also remarkably changed the management of NSCLC^[10]^. The increased expression of programmed cell death protein 1 (PD-1) and programmed cell death ligand 1 (PD-L1) has also been demonstrated to be closely related to KRAS status^[11]^. Controversial results have been reported in multiple clinical studies on the efficacy of immune checkpoints inhibitors (ICIs) in NSCLC with KRAS mutation^[12-15]^. Jeanson *et al.*^[16] ^and Arbour *et al.*^[13] ^demonstrated no difference in response to ICIs among NSCLC patients with or without KRAS mutations. However, other studies have demonstrated more benefits from ICIs for NSCLC patients with KRAS mutation than those without^[14,15]^. In this review, we summarize and analyze the possible mechanisms underlying the resistance to ICIs in NSCLC with KRAS mutation and mainly focus on the role of the tumor microenvironment (TME) and metabolism, therapeutic implications, and potential targets for overcoming the resistance to or improving the efficacy of ICIs treatments in KRAS-mutant NSCLC.

## ICIS TREATMENTS IN NSCLC WITH KRAS MUTATION

Even though the status of KRAS mutation as being able to alter the responses to ICIs in NSCLC has been verified in multiple fundamental studies, solid clinical evidence is still lacking. In this section, we review the clinical trials of ICIs in KRAS-mutant NSCLC patients.

Checkmate-057 is a random and double-blind phase 3 clinical trial with a size of 582 cases, which is designed to compare the efficacy of monotherapy of nivolumab or docetaxel in advanced non-squamous NSCLC patients. Comparing to docetaxel, nivolumab significantly elongates the overall survival (OS) (medium OS = 12.2 months *vs. *9.4 months; HR = 0.73; 95%CI: 0.59-0.89; *P *= 0.0002). The subgroup analysis showed that KRAS-mutant patients have better survival benefits than KRAS-wildtype patients (HR = 0.52, 95%CI: 0.29-0.95; HR = 0.98, 95%CI: 0.29-0.95)^[17]^. Another phase 3 clinical trial, KEYNOTE-042, investigated the efficacy and safety of monotherapy of pembrolizumab or platinum-based chemotherapy in locally advanced or metastatic NSCLC harboring wildtype EGFR/anaplastic lymphoma kinase (ALK) and PD-L1^+^ (TPS ≥ 1%). The results show that the monotherapy of pembrolizumab can prolong the OS compared to platinum-based chemotherapy. Further analysis revealed that KRAS-mutant patients show higher PD-L1 expression and tumor mutational burden (TMB) and have significantly longer progression-free survival (PFS) in the pembrolizumab treatment group than those in the platinum treatment group^[14,18]^. KEYNOTE-189 evaluated the efficacy and safety of pemetrexed and platinum-based therapy or combined with pembrolizumab in metastatic non-squamous NSCLC patients. The results demonstrate that a combination of pembrolizumab and chemotherapy can significantly prolong the OS than chemotherapy regardless of the PD-L1 expression level. Both KRAS-wildtype and -mutant NSCLC patients benefit from chemotherapy combined with pembrolizumab. The pooled analysis of KEYNOTE-189 and KEYNOTE-042 indicated that the non-squamous NSCLC patients with wildtype EGFR/ALK or KRAS show a better prognosis to the combination of pembrolizumab and chemotherapy (HR = 0.55) compared to pembrolizumab monotherapy (HR = 0.86). However, for KRAS-mutant non-squamous patients, the monotherapy of pembrolizumab shows a better prognosis (HR = 0.42 and HR = 0.28) than combination treatment of pembrolizumab and chemotherapy (HR = 0.79 and HR = 1.14)^[19]^. Altogether, the clinical outcomes above indicate that KRAS-mutant NSCLC patients can benefit from immunotherapy or combined treatment of immunotherapy and chemotherapy, whereas the conclusion is not supported by some meta-analyses with more solid evidence. Two meta-analyses and some retrospective analyses have reported that the status of KRAS mutation has no negative association with the survival outcome of immunotherapy in advanced NSCLC patients^[7,16,20,21]^.

One possible illustration is that the co-mutations besides KRAS mutation might play the predictive and prognostic role in response to ICIs in KRAS-mutant NSCLC. Skoulidis *et al.*^[22] ^defined three distinct co-mutation subgroups of the early stage and advanced KRAS-mutant tumors: KRAS^mut^/serine/threonine kinase 11 (STK11)^-/-^, KRAS^mut^/tumor protein 53 (TP53)^-/-^ (KP), and KRAS^mut^/cyclin dependent kinase inhibitor 2A, 2B (CDKN2A, 2B)^-/-^/thyroid transcription factor 1^low ^(KC)^[22]^. The retrospective analysis revealed that the KL patients show less responses to immunotherapy and shorter PFS and OS than KP patients^[23]^. Other investigations have revealed that KP patients show significant benefits from PD-1 blockade monotherapy^[24]^. Collectively, PD-L1 blockade might be more beneficial to KP patients than KL patients.

Taken together, not all the KRAS-mutant NSCLC patients could benefit from ICIs. Co-mutations increase the TMB in patients and might contribute to the poor response to ICIs. The mechanisms of resistance to ICIs in NSCLC with KRAS mutation are sophisticated and not fully investigated, which we discuss in the following sections.

## THE RESISTANT MECHANISMS OF ICIS TREATMENTS IN NSCLC WITH KRAS MUTATION

### KRAS regulates TME through immunomodulatory molecules

Growing evidence has implicated that the intrinsic and extrinsic resistance mechanisms to ICIs might result from the immunosuppressive TME caused by alterations in disparate signaling pathways in tumor cells^[25]^. Previous studies have clearly indicated that the immunosuppressive TME can be regulated by alterations of immunoinhibitors and immunostimulators in tumor and stromal cells^[26]^.

Immune checkpoints exert crucial roles in preventing overreaction and minimize the duration and expansion of immune responses^[27]^. PD-1 (CD279), coded by *pdcd1*, is ubiquitously expressed on various immune cells, including activated T cells, B cells, monocytes, NK cells, and dendritic cells (DCs)^[28]^. PD-1 can be recognized by the ligands, PD-L1 and PD-L2, on normal tissue cells and cancer cells, which maintains the immune homeostasis but might also revoke the anti-tumor immunity^[29]^. After PD-1 binds to PD-L1, PD-1 undergoes a conformational change, which leads to the inactivation of phosphatidylinositol-4,5-bisphosphate 3-kinase catalytic subunit alpha (PI3K)-serine/threonine kinase (AKT) and extracellular signal-regulated kinase 2 (ERK) pathways and, consequently, dysfunction of T cells^[30]^. In addition, studies have reported that tumor cells can secrete exosomes carrying PD-L1, which impede the function of CD8^+^ T cells, CD4^+^ T cells, and Tregs before infiltration^[31]^. It has been widely reported that oncogenic KRAS can increase PD-L1 from different aspects, including transcription, stabilization, and recycling. In LUAD cell lines, KRAS^mut^ has been found to increase the transcriptional level of PD-L1 through the mitogen-activated protein kinase kinase 1 (MEK) pathway^[32]^. In human lung and colorectal cancer, Coelho *et al.*^[33] ^demonstrated that the adenylate-uridylate-rich element-binding protein tristetraprolin (TTP) can bind to the 3’ untranslated region of PD-L1 mRNA and consequently induce the degradation of PD-L1. The oncogenic KRAS activates MEK signaling and further phosphorylates and inhibits the TTP by MK2, which leads to the stabilization of PD-L1 mRNA^[33]^. In addition, it has been reported that mutant KRAS suppresses programmed cell death 4 and then promotes the translation of ADP ribosylation factor 6 and MYCBP associated protein expression, which facilitates PD-L1 recycling driven by platelet-derived growth factor and eventually the immune evasion^[34]^.

As another factor, cytokine-modulated immune responses have been investigated for decades. Cytokines are a type of small proteins secreted by almost all types of cells and can be classified into seven families: interleukin (IL), colony stimulating factor (CSF), interferon (IFN), tumor necrosis factor, tumor growth factor-beta (TGF-β) family, growth factor (GF), and chemokine family^[35]^. Cytokines deliver messages in a paracrine, autocrine, or endocrine manner and can coordinate the recruitment of immune cells, spatial organization, and cellular interactions^[36,37]^. Plentiful cytokines and chemokines have both pro- and anti-inflammatory functions and exert different functions in particular scenarios^[36]^. It has been reported that mutant KRAS can remodel the TME by regulating the production and secretion of pro- or anti-inflammatory cytokines and chemokines^[38]^. The oncogenic mutant KRAS represses the expression of interferon regulatory factor 2 (IRF2) and results in increased production of C-X-C motif chemokine ligand 3 (CXCL3), which binds to C-X-C motif chemokine receptor 2 (CXCR2) on myeloid-derived suppressor cells (MDSCs) and subsequently recruits the MDSCs to impede the cytotoxic T cells^[39]^. In lung cancer, the ubiquitin-specific protease 12 (USP12) induces protein phosphatase 1B, which thwarts transcription factor P65 (NF-κB). NF-κB can promote the production of multiple chemokines, including CXCL8, CXCL1, CCL2, *etc.*, which can result in an immunosuppressive TME through recruiting the M2 macrophages, inducing PD-L1 expression and blocking the responses to T cells. Oncogenic mutant KRAS inhibits USP12 and further promotes the production of chemokines through NF-κB, which ultimately causes the resistance to anti-PD-1^[40]^. Additionally, it has been reported that the development of resistance to anti-PD-L1 and MEK inhibition is the result of increasing IL-17 and IL-22 secreted by accumulated infiltration of Th17 in KRAS^mut^/TP53^-/-^ co-mutant lung cancer patients^[41]^. Moreover, KRAS has been reported to induce the releasing of pro-inflammatory cytokines (IL-6, IL-8, and IL-1) and anti-inflammatory cytokines (IL-10, IL-22, and TGF-β), as reviewed by Hamarsheh *et al*.^[42]^.

**Figure 1:**
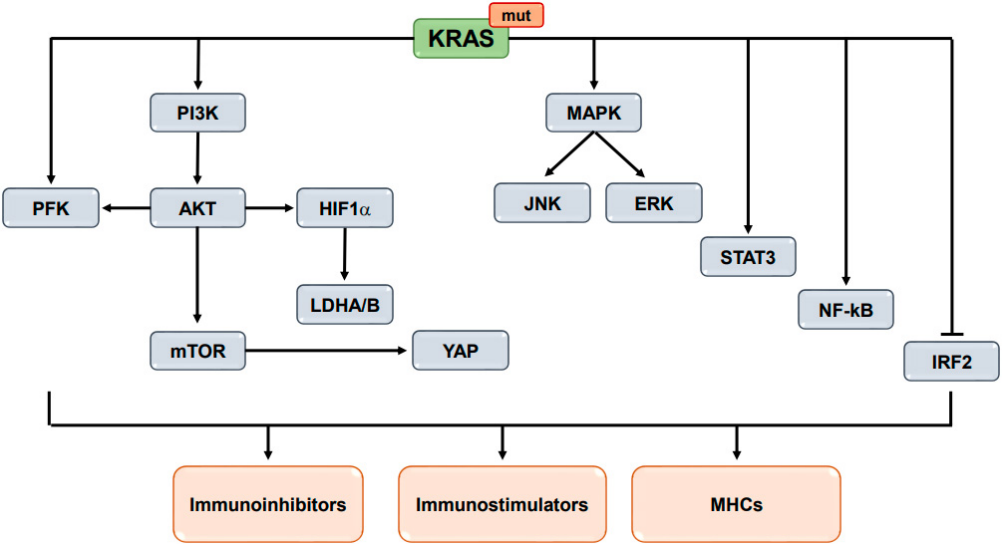
A schematic figure of KRAS-driven signaling pathways in the regulation of immunomodulators. PI3K: Phosphatidylinositol-4,5-bisphosphate 3-kinase catalytic subunit alpha; AKT: serine/threonine kinase; MAPK: mitogen-activated protein kinase 1; ERK: extracellular signal-regulated kinase 2; PFK: phosphofructokinase; HIF1α: hypoxia inducible factor 1 subunit alpha; JNK: mitogen-activated protein kinase 8; mTOR: mechanistic target of rapamycin kinase; LDHA/B: lactate dehydrogenase A/B; YAP: yes1 associated transcriptional regulator; IRF2: interferon regulatory factor 2; STAT3: signal transducer and activator of transcription 3; NF-κB: transcription factor P65; MHCs: major histocompatibility complex.

Immunomodulators are composed of all molecules that can regulate the immune response, including receptors/ligands on both tumor cells and stromal cells in TME and cytokines and chemokines secreted by cells. The studies cited above have uncovered that KRAS could mediate the rearrangement of immunomodulators through different signaling pathway in multiple cancers, which might contribute to the resistance to ICIs in NSCLC. A schematic figure of KRAS-driven signaling pathways in the regulation of immunomodulators is presented in Figure 1.

### KRAS regulates TME through metabolic alteration

For the past decades, many studies have revealed adjustments of energy metabolism in cancers, which are involved in almost every stage of cancer development^[43,44]^. Oncogenic KRAS mutation has been reported to regulate diverse metabolic networks to fulfill the excessive requirement of distinct nutrients to support tumor growth and metastasis^[45,46]^. In this section, we discuss the mechanisms underlying how KRAS orchestrates the TME through metabolic networks.

#### Glycolysis

In normal cells, glucose can be consumed either through oxidative phosphorylation (in mitochondria) or anaerobic glycolysis (in cytosol) to produce energy for cell activities. Glucose is mainly transported into cells with the help of glucose transporters (GLUTs) in an energy-consuming-free way. In cytosol, glucose is catalyzed to glucose-6-phosphate (G6P) by hexokinase (HK). G6P can enter the pentose-phosphorylation pathway with catalyzation of G6P dehydrogenase and be processed to ribulose-5-phosphate for the synthesis of purine. G6P can also be isomerized to fructose-6-phosphate (F6P) and further phosphorylated to fructose-1,6-biphosphate (FBP), which enters the glycolysis pathway. Under the aerobic condition, FBP is catalyzed to pyruvate and then converted to acetyl-CoA by pyruvate dehydrogenase (PDH), which participates in the TCA cycle. Under the anaerobic condition, pyruvate does not enter the TCA cycle but is converted to lactate by the lactate hydrogenase (LDH). Under normal condition, a small portion of F6P also enters the hexosamine pathway (HBP) with the help of glutamine--fructose-6-phosphate aminotransferase (GFPT) and provides glycans for protein glycosylation^[47,48]^.

Back in 1985, it was reported that rat-1 cell maintains a higher level of glycolysis in the presence of *ras *transfection^[49]^. Accumulating evidence shows that oncogenic KRAS mutation modulates glycolysis through promoting the uptake of glucose and increasing glycolysis flux. In pancreatic ductal adenocarcinoma (PDAC) with KRAS^G12D^, metabolomic and RT-qPCR analyses have revealed increasing glucose transporter solute carrier family 2-member 1 (GLUT1) expression and enhanced glycolysis flux. KRAS^G12D^ was found to upregulate the expression of several enzymes, including HK, PFK, enolase, *etc.*^[50]^. Many studies have explored in more detail about how mutant KRAS interacts with the enzymes involved in the carbon metabolism pathways. In KRAS-driven PDAC, 6-phosphofructo-2-kinase/fructose-2,6-biphosphatase 3 is phosphorylated by p38γ induced by KRAS mutation and forms a ternary complex with solute carrier family 2 member 2 (GLUT2), which promotes the uptake of glucose^[51]^. In addition, GLUT1 has been found significantly upregulated in NSCLC with KRAS mutation^[52]^. KRAS4A, a shorter isoform of KRAS, has been found to bind to the N-terminal of HK1 and block the allosteric inhibition feedback, which promotes the transition of glucose to G6P and increases the glycolysis flux^[53]^. Based on both an *in vivo* mice model and an *in vitro* H23 cell line study, Wang *et al.*^[54]^ reported that KRAS activation is required for HK2 expression. In a KRAS^mut^/STK11^-/-^ co-mutated lung cancer model, KRAS mutation induces the expression of GLUT. Meanwhile, the loss of STK11 impedes the phosphorylation and activation of protein kinase AMP-activated catalytic subunit alpha 1 (AMPK), thereby leading to the activation of GFPT enzyme. The increasing activity of GLUT and GFPT promotes HBP^[55]^. Glucose is also the primary energy source for the proliferation and differentiation of immune cells^[56,57]^. As a result of increasing glycolysis in tumor cells, the access to glucose for immune cells is limited in TME. A cruel competition between tumor cells stromal cells consequently impairs the glycolytic metabolism and normal functions of immune cells^[58,59]^. More specifically, the limited glycolysis causes the weakened activity of the AKT/mammalian target of rapamycin (mTOR) pathway in T cells, which impedes the production of INF-γ^[59]^. The limited availability of glucose also results in insufficient production of glycolytic metabolite phosphoenolpyruvate to maintain the anti-tumor activity through calcium and NFAT signaling pathways in infiltrated T cells^[60]^. In NK cells, the aberrant expression of fructose-1,6-bisphosphatase (FBP1) in gluconeogenesis and TCA cycle leads to the halt in glycolysis and induction in cell death^[61]^. It has also been found that inhibition of glycolysis impairs the oligomerization of C-C motif chemokine receptor 7 and thus hinders the migration of DCs to draining lymph nodes^[62]^.

One important metabolite of glycolysis is lactate. In KRAS-driven NSCLC, the high expressions of LDH-A and LDH-B are reported to be associated with tumorigenesis and disease progression^[63,64]^. One study found that phosphoglycerate kinase 1 (PGK1) can be phosphorylated by ERK1/2, which is the downstream of KRAS. The phosphorylated PGK1 phosphorylates PDHK1, the latter further phosphorylating and inhibiting PDH, which blocks the conversion of pyruvate to acetyl-CoA and subsequently promotes the conversion of pyruvate to lactate^[65]^. In PDAC, hypoxia and active KRAS can induce the expression of hypoxia inducible factor 1 subunit alpha (HIF1α) and inhibit the degradation of HIF1α, respectively. HIF1α then transcriptionally enhances the expression of the lactate transporter, solute carrier family 16 member 4 (MCT4), which transports lactate out of cells^[66]^. The accumulation of lactate from the excessive glycolysis in tumor cells is transported by MCT4 out of cells, which generates a low-pH environment that discourages the normal proliferation and function of immune cells^[67]^. The accumulated lactate promotes the polarization of macrophages toward anti-inflammatory M2 phenotype and leads to a downregulation of interferon gamma (IFN-γ) secreted by the infiltration of T cells and NK cells^[68,69]^. Besides, the accumulated lactate directly blocks the glycolytic flux in T cells^[70]^.

Taken together, the studies described above suggest that KRAS mutation can promote glycolysis by promoting various signaling pathways and molecules, such as related enzymes and transporters. The outrageous requirement of glucose and excessive excretion of lactate impair the normal proliferation and function of different immune cells, which ultimately diminishes the anti-tumor activity.

#### Lipid metabolism

Lipid metabolism is constantly in homeostasis due to the balance of fatty acids synthesis (FAS) and fatty acids oxidation (FAO) in normal cells. In the process of *de novo* lipogenesis, citrate is catalyzed to acetyl-CoA in assistance with ATP-citrate lyase (ACLY) and then transformed to palmitate by FA synthetase (FASN), phosphatidic acids, and finally triacylglycerol. Triacylglycerol can be stored in the lipid droplets (LDs) in the cytoplasm^[71]^. Phospholipids are another source of lipogenesis and can be converted to arachidonic acid (ARA) by phospholipases (PLA2)^[72]^. ARA can be catalyzed by cyclooxygenase 1 and 2 (COX-1 and COX-2) and transformed to prostaglandins 2 (PGE2), which has been shown to play a vital role in tumorigenesis and immune response^[73]^. The FAO process can be classified into harmless β-oxidation and fatal lipid peroxidation. In β-oxidation, FAs are catalyzed to produce acetyl-CoA, NADH, and FAD. Besides, lipid peroxidation is believed to start from polyunsaturated fatty acid chains, induced by reactive oxygen species (ROS), and results in ferroptosis^[74]^. In the mouse model of KRAS-driven LUAD, the loss of stress-induced metabolic regulators, regulated in development and DNA damage responses 1, can induce a HIF-dependent lipid storage pathway, which induces the FA transporter CD36 and thereby uptake of exogenous fatty acid^[75]^. KRAS mutation has been shown to promote FA synthesis through inducing FASN and stearoyl-CoA desaturase mediated by ERK2^[76]^. Besides, the FA metabolism has been reported to be upregulated by the PI3K-mTOR pathway^[77]^, which has already been demonstrated to be regulated by KRAS^[78]^. The active AKT can facilitate both the ACLY and mTORc1. ACLY promotes the transformation of citrate to acetyl-CoA, which subsequently boosts lipogenesis. Active mTORc1 can activate slicing factor serine/threonine-protein kinase (SRPK2) and transcriptional factor sterol regulatory element binding protein-1c in the nucleus, which ensures the expression of a series of lipogenesis enzymes, including ACLY, FASN, *etc*.^[79-81]^. Besides promoting FAS, KRAS mutation has also been reported to suppress the oxidation of lipids. The suppression of hormone-sensitive lipase (HSL) by KRAS mutation leads to the decreased β-oxidation of FAs, which results in the accumulation of FAs^[82]^. STK11 deficiency has been found to cause the downregulation of FAO upregulation of fatty acid synthesis via activation of acetyl-CoA carboxylase due to the low level of phosphorylation of AMPK^[83]^. Kelch like ECH associated protein 1 (KEAP1) is another frequently observed co-mutation along with KRAS^mut^/STK11^-/-^. KEAP1 mutation has been reported to decrease lipid peroxides via enhancing the transcription factor nuclear factor, erythroid 2 like 2, which directly upregulates the suppressor of ROS, glutathione peroxidase 4^[74,84,85]^. The accumulated FAs in the TME have been found to cause the dysfunction of immune cells in KRAS-mutant mice model^[86]^. In the infiltrated cytotoxic CD8^+^ T cells, the long-chain fatty acids are unbreakable and can result in a reduction of very-long-chain acyl-CoA dehydrogenase and lipid toxicity in T cells, which cause the exhaustion of infiltrated T cells^[87]^. In addition, the excess FAs caused by immunoglobulin-like transcript 4/paired immunoglobulin-like receptor B via mitogen-activated protein kinase 1/ERK1/2 signaling has been demonstrated to cause the senescence of effector T cells^[88]^. Moreover, the absorption of the FA-carrying tumor-derived exosomes by DCs can generate increasing storage of FAs and the enhancement of β-oxidation. The shift of mitochondrial oxidative phosphorylation due to the fuel alteration results in the dysfunction of DCs^[89]^.

Previous studies have revealed that the accumulated FAs provides sufficient raw material for PGE2 production^[90,91]^, and KRAS mutation can also enhance the production of PGE2 via upregulation of COX-2^[92]^. In NSCLC, PGE2 has been found to induce forkhead box P3 (FOXP3) expression and subsequently promotes the activity of CD4^+^ and CD25^+^ Tregs^[93]^. In metastatic murine renal carcinoma, overproduced PGE2 has also been reported to suppress the anti-tumor cytotoxic T cell lymphocyte responses by preventing the production of IFN-γ^[94]^. Moreover, the excess PGE2 in TME suppresses the production of X-C motif chemokine ligand 1 and CCL5 by NK cells and further blocks the recruitment of the conventional type 1 dendritic cells (cDC1s) through downregulating the respective receptors on cDC1s^[95]^. Increasing evidence indicates that the accumulated PGE2 in tumor cells exerts an immunosuppressive TME.

Cholesterol is normally synthesized from acetyl-CoA. Recent studies have found that cholesterol exerts an immunosuppressive TME by promoting the expression of immune checkpoint on CD8^+^ T cells, which induces ER stress and consequently exhaustion of those T cells^[96,97]^. In KRAS-driven NSCLC, the synthesis of cholesterol has been found to be induced by Myc proto-oncogene protein (MYC) activation. The activation of MYC leads to an accumulation of cholesteryl esters (CE) stored in LDs and the increased influx and the decreased efflux of cholesterol in tumor cells. Of note, the deactivation of MYC following activation gives rise to an additional increase of CEs^[98]^.

In conclusion, there is supporting evidence indicating that FA metabolism can be induced by KRAS mutation through regulating various molecules. The excessive synthesis of FA and associated metabolites impedes the normal function of immune cells.

#### Glutamine and tryptophan metabolism

In normal cells, amino acid metabolism facilitates cells with energy and material for the biosynthesis of macromolecules, such as proteins and nucleotides. Amino acid metabolism reprogramming is required for hyperactive tumor cells to meet the high demand of energy and proteins required for cell proliferation^[99]^. Among the 21 amino acids within the human body, glutamine and tryptophan have been broadly reported to be rewired in various tumor cells and build an immune suppressive TME, which facilitates tumor cells to evade immune surveillance.

Glutamine can be successively transformed to glutamate, α-ketoglutarate, oxaloacetate, and aspartate by the catalyzation of glutaminase (GLS1), glutamate dehydrogenase 1, and glutamic-oxaloacetic transaminase 1/2, respectively^[100]^. KRAS mutation in NSCLC enhances the glutamine metabolism by inducing glutamine uptake, which results in a glutamine deficiency in TME^[101]^. KRAS mutation has been found to induce multiple glutamine transporters, such as solute carrier family 1 member 5 (SLC1A5) in NSCLC^[102,103]^, solute carrier family 7 member 5 (SLC7A5)^[104]^, and solute carrier family 38 member 2 (SLC38A2) via activation of hippo effector yes1 associated transcriptional regulator (YAP1)^[103]^. The upregulated transporter SLC1 increases the influx of glutamine, and the increased antiporter SLC7A effluxes glutamines and intakes other essential amino acids. The essential amino acids transported by SLC7A induce mTORC1-ribosomal protein S6 kinase B1 signaling and further promote protein synthesis^[103,104]^. It has also been shown that the mRNA level of GLS is higher in KRAS-mutated NSCLC, which indicates a higher consumption of glutamine in NSCLC^[102]^. Additionally, the KRAS^mut^/STK11^-/-^/KEAP1^-/-^ co-mutant NSCLC was found dependent on glutaminolysis for fuel and was specifically sensitive to GLS inhibitors^[85]^. The high uptake of glutamine in tumor cells leads to a glutamine deficiency in TME, which causes immune suppression through blocking active T cells or producing immune suppressive cells. One study illustrated that the lack of glutamine results in the increase of HIF and then the rising secretion of IL-23 by macrophages, which promotes the proliferation of Tregs through the STAT3 signaling pathway. Tregs subsequently secretes TGF-β and IL-10 and suppresses the cytotoxicity of T cells^[105]^. Besides the shortage of glutamine in the TME, other conditions can also result in glutamine deprivation in tumor infiltrated immune cells. One study showed that the shortage of glucose causes defective N-linked glycosylation and endoplasmic reticulum stress and accordingly activates the endoplasmic reticulum to nucleus signaling 1 (IRE1) - X-box binding protein 1 (XBP1) axis. Activated XBP1 represses the glutamine transporters through post-translational regulation, which blocks glutamine uptake. The low level of glucose and glutamine causes the dysfunction of mitochondria, which impedes the production of IFN-γ by active T cells^[106]^. Moreover, glutamine deprivation increases the secretion of G-CSF and GM-CSF through activating the IRE1α/c-Jun N-terminal kinase pathway in cancer cells, which manipulates the formation of immunosuppressive MDSCs in TME^[107]^. However, the results from one study also indicate that high glutamine in TME tends to polarize the macrophages to pro-tumorigenic M2 type and inhibit the differentiation of the M1 macrophage^[108]^, which is contrary to the conclusion from the above research. The contradictory conclusion of deprived glutamine indicates the complexity of glutamine in regulating TME.

Tryptophan can either be catalyzed to 5-hydrooxytryptophan, serotonin, and 5-hydroxyindoleacetic acid by tryptophan hydroxylase 1 and 2 (TPH1, TPH2), dopa decarboxylase, and monoamine oxidase A and B, respectively, or be catalyzed to kynurenine, kynurenic acid by indoleamine 2,3-dioxygenase 1 and 2 (IDO1/2), and tryptophan 2,3-dioxygenase (TDO2). In NSCLC, the KRAS mutation has been shown to reprogram tryptophan metabolism through upregulating the expression of immune checkpoint markers IDO1 according to microarray data^[109]^. The detailed mechanism remains to be discovered. The tryptophan metabolism has a pivotal role in promoting immune evasion. In tumor cells, tryptophan is transformed into kynurenine by IDO1. The upregulated IDO1 level induces excess kynurenine, which could be transferred to TME to promote Tregs and suppress effector T cells^[110]^. It has been illustrated that the activity of IDO can reflect the advanced disease, tumor metastasis, and immunotherapy responses to PD-1 inhibitors. The high expression of IDO can recruit immunosuppressive MDSCs through Tregs, which is Foxp3 dependent^[111,112]^.

In summary, cancer cells can rely on glutamine as the main source of fuel and the precursor of other biomacromolecules. The deficiency of glutamine and increase of metabolite from tryptophan metabolism in TME give rise to an immunosuppressive phenotype. KRAS has been found to modulate several signaling pathways involved in glutamine metabolism, whereas further work is required to illustrate the more detailed mechanisms.

**Figure 2:**
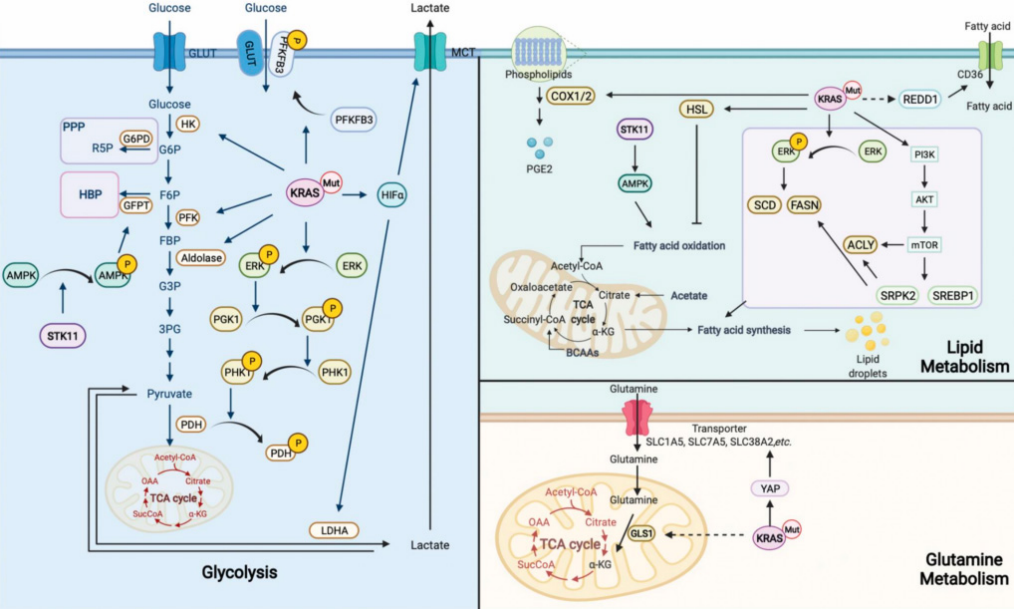
A schematic figure of KRAS-driven signaling pathways in the regulation of metabolic signaling pathways. GLUT: Glucose transporters; HK: hexokinase; PFK: phosphofructokinase; PI3K: phosphatidylinositol-4,5-bisphosphate 3-kinase catalytic subunit alpha; AKT: serine/threonine kinase; MAPK: mitogen-activated protein kinase 1; ERK: extracellular signal-regulated kinase 2; PDH: pyruvate dehydrogenase; PGK: phosphoglycerate kinase 1; HIF1α: hypoxia inducible factor 1 subunit alpha; JNK: mitogen-activated protein kinase 8; mTOR: mechanistic target of rapamycin kinase; LDHA/B: lactate dehydrogenase A/B; YAP: yes1 associated transcriptional regulator; IRF2: interferon regulatory factor 2; STAT3: signal transducer and activator of transcription 3; NF-κB: transcription factor P65; HSL: hormone-sensitive lipase; COX: cyclooxygenase; SCD: stearoyl-CoA desaturase; FASN: FA synthetase; ACLY: ATP-citrate lyase; GLS: glutaminase.

Besides the metabolism mentioned above, mutant KRAS has also been reported to regulate additional metabolic signaling pathways, including asparagine metabolism^[113]^, authophagy^[46,114]^, mcropinocytosis^[115]^, *etc*. More detailed reviews of the metabolic rewiring driven by KRAS mutation can be found elsewhere^[46,116-118]^. Nutrient scavenging is a practical method for both tumor cells and immune cells to grab necessities from TME. Tumor cells and immune cells often outcompete stromal cells for nutrients or metabolites in TME, which leads to a nutrient-deprived environment^[43]^. However, most nutrients and some metabolites are also essential for the biosynthesis of macromolecules and production of energy craved by immunocytes^[56,57,61,119,120]^. Studies have also asserted that excess metabolites, such as lactate, alter the environment and affect the functions of immune cells^[60,86,87,89,121]^. A more comprehensive description of cancer metabolic reprogramming and immune response can be found in other reviews^[122-125]^. Collectively, the limited nutrients and redundant metabolites arouse the dysfunction in immune cells, which is one of the leading causes of resistance to ICIs. Taken together, KRAS mutation rewires the metabolic network in TME and thereby might cause a poor response to ICIs. A schematic figure of KRAS-driven metabolic signaling pathways in cancer cells is shown in Figure 2.

## PROSPECTIVE STRATEGIES FOR OVERCOMING RESISTANCE TO ICIS

According to the ASCO guidelines, five main therapeutic strategies are applied in NSCLC: surgery, radiation therapy, chemotherapy, target therapy, and immunotherapy. The therapeutic schedule depends mostly on the stage and mutation type. As illustrated above, the efficacy of monotreatment of immunotherapy is controversial in NSCLC patients; thus, researchers have begun investigating the combinational strategy of immunotherapy and other therapies. In this section, we discuss the promising combined treatments of immunotherapy and other therapies in preclinical and clinical studies. 

It is discussed above that PD-1/-L1 expression plays an essential role in response to ICIs. Therefore, targeting pathways modulating PD-1/-L1 expression might show potential for overcoming the resistance to ICIs, which has been demonstrated in several studies. In the KRAS-mutant lung cancer model, blockade of both PD-1 and helix-loop-helix transcription factor inhibitor of differentiation 1 knock out significantly enhances the amount of CD8^+^ T cells as well as the expression of PD-L1, which impairs the tumor growth and increases the survival^[126]^. In other studies of KRAS^mut^ lung cancer, anti-PD-1 combined with inhibition of the AKT-mTOR pathway by mTOR inhibitor or the STAT3 pathway by natural compound luteolin can remarkably decreased the expression of PD-1 or PD-L1, respectively, which consequently surmounts the resistance to anti-PD-1^[127,128]^.

Other potential strategies to overcome the ICIs resistance include promoting the proliferation and activation of cytotoxic T cells and thwarting immunosuppressive immune cells, among others. The cholesterol-lowering drug statin has been reported to inhibit prenylation of KRAS and cause ER stress and immunogenic cell death, which cross-primes DCs and provokes CD8^+^ T cells. Combinational treatment of anti-PD-1, statin, and oxaliplatin has shown the potential to overcome resistance to anti-PD-1^[129]^. In the KRAS^mut^/TP53^-/-^ lung cancer mouse model, chemokine CCL7 has also been found to recruit CD11c^+^ or Zbtb46^+^ DCs and promote T cell expansion, which increases the immune responses. When combined with anti-PD-1, CCL7 profoundly increases the survival of the NSCLC mouse model^[130]^. The BET bromodomain inhibitor has also been demonstrated to increase the number of infiltrated T-helper type 1 cells and reduce the population of Tregs, which augments the anti-tumor response to anti-PD-1^[131]^. Utilization of antibodies or natural compounds to block immunosuppressive cytokines or chemokines has been reported to hamper the resistance to ICIs in KRAS-mutant NSCLC mouse models^[41,132]^.

Due to the approval of the KRAS^G12C^ inhibitor AMG 510 in KRAS^G12C^ NSCLC by the FDA, studies have been investigating the effect of the combination of KRAS inhibitors and ICIs at the bench and in clinics. In the KRAS^G12C^ CT26 syngeneic mouse model, AMG510 treatment has been found to induce a pro-inflammatory microenvironment by enhancing the tumor infiltrated CD8^+^ T cells, DCs, NKs, *etc*., and it exerted extended anti-tumor efficacy when combined with anti-PD-1^[133]^. A phase 1 clinical study designed to evaluate the safety and efficacy of AMG 510 and immunotherapy is in process now (NCT03600883). In the KRAS^G12C^ CT26 syngeneic mouse model, KRAS inhibitor MRTX849 combined with anti-PD-1 has also been shown to increase major histocompatibility complex I expression and decrease immunosuppressive factors, which increased PFS compared to monotherapy^[134]^. Phase 1/2 studies evaluating the safety, tolerability, PK, and clinical activity of MRTX849 in combination with pembrolizumab in patients with NSCLC are now recruiting (NCT04613596 and NCT03785249).

Other attempts have been made to combine ICIs with inhibitors targeting KRAS downstream. A phase 1/2 clinical trial has investigated the efficacy of the PLK inhibitor rigosertib combined with nivolumab in NSCLC patients with KRAS mutation (NCT04263090). The early results indicate that 29% (2/7) of patients achieved partial responses, most of whom harbor a KRAS^G12C^ or KRAS^G12V^ mutation; 43% (3/7) of patients had a stable disease; and 57% (4/7) of patients had progressive disease^[135]^. Studies on the combinational efficacy of other KRAS^G12C^ inhibitors and ICIs, such as GDC-6036 (NCT04449874) and JDQ-443 (NCT04699188), are now recruiting. The efficacy of ICIs combined with tumor vaccine mRNA-5671, which specifically target KRAS^G12C/D/V^ and KRAS^G13D^, is now under investigation (NCT03948763).

Combination treatments which are not specifically for KRAS-mutant NSCLC have also received positive results in the NSCLC group. Multiple ICI treatment has shown promising results in NSCLC patients. In a phase 3 trial, patients were designed to receive mono-nivolumab, nivolumab combined with ipilimumab, nivolumab combined platinum-based chemotherapy, or mono-chemotherapy. The nivolumab combined with ipilimumab treatment group (medium OS = 17.1 months; 95%CI: 15.0-20.1) has shown longer median duration of overall survival compared to the chemotherapy treatment group (medium OS = 14.9 months; 95%CI: 12.7-16.7) among patients with PD-L1 (TPS ≥ 1%). The two-year overall survival rates were 40% and 32.8% and the median duration responses were 23.2 and 6.2 months, in the nivolumab combined with ipilimumab treatment group and chemotherapy treatment group, respectively. Patients with a PD-L1 (TPS < 1%) also benefit from nivolumab combined with ipilimumab (medium OS = 17.2; 95%CI: 12.8-22.0) compared with chemotherapy treatment (medium OS = 12.2 months; 95%CI: 9.2-14.3)^[136]^. In a phase 2 clinical trial, 97 patients were enrolled to study whether stereotactic body radiotherapy (SBRT) enhances the tumor responses to pembrolizumab. The results show that the ORR at 12 weeks was 36% in experimental arm (SBRT plus pembrolizumab) *vs.* 18% in control arm (pembrolizumab), the median PFS was 6.6 months (SBRT plus pembrolizumab, 95%CI: 4.0-14.6) *vs.* 1.9 months (pembrolizumab, 95%CI: 1.7-6.9), and the median OS was 15.9 months (SBRT plus pembrolizumab, 95%CI: 7.1 to not reached) *vs. *7.6 months (pembrolizumab, 95%CI: 6.0-13.9)^[137]^. A phase 2 trial enrolled 96 patients to study the efficacy of combinational anti-PD-1 (SHR-1210) and anti-VEGFR (Apatinib) in NSCLC patients with wild-type EGFR and ALK (NCT03083041). The ORR was 29.7% and DCR was 81.3%. Further analysis showed that the ORR reached 50% in the patients with high TMB, indicating that this combo-treatment is acceptable in NSCLC patients with high TMB^[138]^.

All the clinical trials mentioned above have illustrated good outcomes comparing with mono-immunotherapy in NSCLC, regardless of KRAS status. Further umbrella analyses are still needed to elucidate the combo-treatment efficacy on the KRAS-mutant NSCLC groups. All clinical trials are listed in Table 1.

**Table 1:** Immune checkpoint inhibitors active in clinical trials

**Immune checkpoint inhibitors**	**NCT identifier numbers**	**Combined therapy**	**Target**	**Allocation**	**Phase**	**Size**	**Primary ends**	**Status**
**Anti-PD-1/-L1**	NCT03600883	AMG 510	KRAS^G12C^	Randomized	Phase 1	733	AEs, DLTs, significant clinical changes	Recruiting
**Atezolizumab**	NCT04449874	GDC-6036 bevacizumab, cetuximab, rrlotinib	KRAS^G12C^ VGFR EGFR TKI	Non-randomized	Phase 1	236	AEs, DLTs	Recruiting
**Pembrolizumab**	NCT04429542	BCA101	EGFR/TGF-β	Non-randomized	Phase 1	292	Safety, Cmax, PFS, AEs, ORR, OS, DLTs, MTD	Recruiting
	NCT04613596	MRTX849	KRAS^G12D^	N/A	Phase 2	120	Clinical activity	Recruiting
	NCT03785249	MRTX849	KRAS^G12D^	N/A	Phase 1	565	Safety, tolerability, drug levels, molecular effects, and clinical activity	Recruiting
	NCT03299088	Trametinib	MEK	Non-randomized	Phase 1	15	DLTs, ORR, PFS	Active, not recruiting
	NCT02779751	Abemaciclib Anastrozole	CDK Estrogen synthesis	Non-randomized	Phase 1	100	PFS, AEs, ORR, OS, DCR, PK	Active, not recruiting
	NCT03948763	mRNA-5671/V941	Vaccine	Non-randomized	Phase 1	100	DLTs, AEs, ORR	Recruiting
	NCT04340882	Docetaxel Ramucirumab	Chemotherapy VEGFR	N/A	Phase 2	41	PFS, AEs, ORR, OS	Recruiting
	NCT03225664	Trametinib	MEK	N/A	Phase 1/2	37	ORR	Active, not recruiting
	NCT02492568	Radiation		N/A	Phase 2	96	ORR, toxicity	Complete
**Nivolumab**	NCT04263090	Rigosertib	PLK1	N/A	Phase 1/2	30	MTD, ORR, PFS, OS	Recruiting
	NCT02852083	Pioglitazone Clarithromycin	PPARγ Bacteria proteins	Randomized	Phase 2	86	PFS, AEs, ORR, OS	Unknown status
	NCT02492568	Ipilimumab	Anti-CTLA-4	N/A	Phase 3	1980	OS, ORR, disease related symptom	Recruiting
SHR-1210	NCT03083041	Apatinib	Anti-VEGFR	N/A	Phase 2	117	AEs, ORR	N/A

AEs: Adverse events; DLTs: dose-limiting toxicities; PFS: progression-free survival; OR: overall survival; RR: response rate; MTD: maximum tolerant doses; ORR: objective response rate; DCR: disease control rate; PK: pharmacokinetics; Cmax: plasma concentration.

The application of dietary modifications to supplement other cancer therapies is drawing more attention currently. The intake of different nutrients might alter the nutrient availability in the plasma, thus the TME^[139]^. Preclinical and retrospective studies have assumed that cancer development and prognosis are negatively correlated to unhealthy diets, such as diets high in sodium and fat^[121,140]^. Growing research has found that restriction or supplementation of specific metabolites might ameliorate therapeutic responses. The manipulation of nutrient accessibility remarkably reprograms the metabolic activity and therefore leads to alterations in cell activities and sensitivity to therapies, which is mainly modulated by nutrient-sensing pathways^[141-144]^. The dietary modifications enhance cancer therapy through sophisticated mechanisms, which are well summarized in other reviews^[145-147]^. As mentioned above, glucose is the primary source for not only energy but also metabolic intermediates for macromolecule synthesis for both tumor cells and immune cells. A diet low in glucose but normal in calories can reduce the blood glucose and decelerate tumor growth in some tumor models^[148]^. However, the glucose restriction might also affect the normal function of immune effect cells. During the process of proliferation, differentiation, and activation of immune cells, the requirement for glucose might differ. Studies have found that inhibition of glycolysis restrains the differentiation of CD4^+^ T cells to Treg cells and promotes the differentiation of activated CD8^+^ T cells to long-lived memory CD8^+^ T cells^[119,149]^. Accordingly, we presume that timing and personalized diet should be taken into account when applying glucose restriction to supplement ICI treatment. Likewise, the lipid and amino acid metabolisms function differently at different stages of differentiation and activation of immune cells, thus the need for nutrients varies^[120,150-152]^. Therefore, it is critical to understand the metabolic network and landscape in TME in KRAS-driven NSCLC and how they affect the behavior of tumor cells and the function of stromal cells. Moreover, the impact of dietary modification on ICI response might be influenced by patient-specific variables. Many studies are currently investigating the molecular mechanisms and evaluating the effects of dietary interventions in enhancing cancer therapies. Collectively, dietary modification is a promising strategy for overcoming resistance to or improving the efficacy of ICI treatment in KRAS-mutant NSCLC.

## CONCLUSION

Further understanding of the resistance mechanisms to ICIs mediated by KRAS mutation in NSCLC could provide implications on prospective therapeutic interventions to overcome the resistance or improve the efficacy. This will need further investigations to unearth metabolic pathways regulated by specific KRAS mutations, as well the modulatory effect on shaping TME directly and indirectly. Additional attempts to identify metabolic signaling pathways that promote immunosuppressive TME and resistance to ICIs will help discover the targetable metabolic vulnerabilities to improve the efficacy or overcome the resistance to ICIs through dietary modifications.

## DECLARATIONS

### Copyright

© The Author(s) 2022.
